# A molecular signature of normal breast epithelial and stromal cells from Li-Fraumeni syndrome mutation carriers

**DOI:** 10.18632/oncotarget.175

**Published:** 2010-10-06

**Authors:** Brittney-Shea Herbert, Rebecca A. Chanoux, Yunlong Liu, Peter H. Baenziger, Chirayu P. Goswami, Jeanette N. McClintick, Howard J. Edenberg, Robert E. Pennington, Steven M. Lipkin, Levy Kopelovich

**Affiliations:** ^1^ Department of Medical and Molecular Genetics, Indiana University School of Medicine, Indianapolis, IN, USA; ^2^ Indiana University Melvin and Bren Simon Cancer Center, Indiana University School of Medicine, Indianapolis, IN, USA; ^3^ Center for Computational Biology and Bioinformatics, Indiana University School of Medicine, Indianapolis, IN, USA; ^4^ Division of Biostatistics, Department of Medicine, Indiana University School of Medicine, Indianapolis, IN, USA; ^5^ Center for Medical Genomics, Indiana University School of Medicine, Indianapolis, IN, USA; ^6^ Department of Biochemistry and Molecular Biology, Indiana University School of Medicine, Indianapolis, IN, USA; ^7^ Department of Surgery, Indiana University School of Medicine, Indianapolis, IN, USA; ^8^ Departments of Medicine and Genetic Medicine, Weill Cornell Medical College, New York, NY, USA; ^9^ Division of Cancer Prevention, National Cancer Institute, Bethesda, MD, USA

**Keywords:** one-hit effects, gene expression profiling, Li-Fraumeni Syndrome, chemoprevention, breast cancer

## Abstract

Specific changes in gene expression during cancer initiation should enable discovery of biomarkers for risk assessment, early detection and targets for chemoprevention. It has been previously demonstrated that altered mRNA and proteome signatures of morphologically normal cells bearing a single inherited “hit” in a tumor suppressor gene parallel many changes observed in the corresponding sporadic cancer. Here, we report on the global gene expression profile of morphologically normal, cultured primary breast epithelial and stromal cells from Li-Fraumeni syndrome (LFS) *TP53* mutation carriers. Our analyses identified multiple changes in gene expression in both morphologically normal breast epithelial and stromal cells associated with *TP53* haploinsufficiency, as well as interlocking pathways. Notably, a dysregulated p53 signaling pathway was readily detectable. Pharmacological intervention with the p53 rescue compounds CP-31398 and PRIMA-1 provided further evidence in support of the central role of p53 in affecting these changes in LFS cells and treatment for this cancer. Because loss of signaling mediated by *TP53* is associated with the development and survival of many human tumors, identification of gene expression profiles in morphologically normal cells that carry “one-hit” p53 mutations may reveal novel biomarkers, enabling the discovery of potential targets for chemoprevention of sporadic tumors as well.

## INTRODUCTION

Germline *TP53* mutations occur in Li-Fraumeni syndrome (LFS), a rare, inherited autosomal dominant disorder which is characterized by early onset of multiple primary tumors [1]. These malignancies include sarcomas, breast cancers, glioblastomas, adrenal cortical tumors, colon cancers, lung cancers, and leukemias, among others [1-2]. Mutations in the *TP53* tumor suppressor gene are found in 70% of classic LFS families and 30% of LFS-like kindreds [3-4]. Germline mutations occur in one allele and, as predicted by the classic Knudson two-hit hypothesis, the second allele is somatically inactivated through mutation, deletion or epigenetic repression (i.e., loss of heterozygosity, LOH) in LFS mutation carrier cancers [4-5]. The two-hit hypothesis has been validated recently through recent findings of one-hit effects in cancer [6-11].

Clinical criteria for diagnosis are established for classic LFS. These criteria include individuals with an early onset sarcoma, a first degree relative with cancer before age 45 and another first-degree relative with sarcoma at any age or any cancer before age 45 [1]. Furthermore, the National Comprehensive Cancer Network (NCCN) guidelines provide recommendations for screening LFS family members for early detection. These recommendations include annual dermatological and neurological exam, colonoscopy every 2-5 years, breast MRI beginning at age 20, and family specific studies (NCCN; www.nccn.org).

Despite the significant susceptibility to cancer, breast cancer in particular, and risk of early death in LFS families, there are currently no molecular approaches for risk assessment or early detection, nor cancer chemoprevention strategies to help LFS families. Molecular diagnosis of LFS is complicated by the fact that almost all LFS-associated *TP53* mutations are missense [12-13]. Missense variants are difficult to classify distinctly as deleterious or benign due to the high level of evidence required for clinical diagnosis. Clinicians are often reluctant to make a diagnosis of LFS because of the inherent ambiguity of classifying missense variants. Therefore, molecular diagnostics that use different approaches to confirm and validate diagnosis of LFS in individuals who carry *TP53* missense mutations are needed. These diagnostics are also useful in patients in whom no identifiable mutation is found (i.e., false negatives). Because tumors arise in multiple stages, there are several potential steps at which tumor initiation or progression could be targeted to prevent malignancies.

Here, we describe whole genome expression profiling of primary epithelial and stromal cells from LFS patients with defined germline *TP53* mutations and paired normal cell samples processed in parallel. We demonstrate that the morphologically normal epithelial and stromal cells from LFS mutation carriers display altered gene expression profiles in a cell type-specific manner. Notably, in breast epithelial and stromal fibroblast cells with TP53 haploinsufficiency, a dysregulated p53 signaling pathway was readily detectable using gene expression profiling technology. The abnormal alterations seen in LFS cells are distinct from previous FAP and BRCA1-2 specific gene expression changes [8, 10]. While gene expression profiling is currently used to analyze breast cancers and assess recurrence risk and chemosensitivity (such as with Oncotype Dx or Mammaprint), it has not yet been applied to the detection of morphologically normal, but cancer susceptible, tissues. Our study shows that gene expression profiling is able to distinguish *TP53* haploinsufficient breast epithelial and stromal cells from matched tissue from an individual with wild-type *TP53*. These data suggest that genomic profiling can help define molecular targets for chemoprevention and biomarkers of breast cancer risk impacted by early alteration in *TP53*. Significantly, pharmacological intervention with the p53 rescue compounds CP-31398 and PRIMA-1 provided further evidence in support of the central role of p53 in affecting these changes in LFS cells and treatment for this cancer. These studies will provide more precise molecular markers specific for early *TP53* alterations and enable mechanism-based early detection and personalized prevention strategies for cancer.

## RESULTS

### Gene expression profiling of single-hit LFS epithelial and stromal cell cultures.

**Figure 1:**
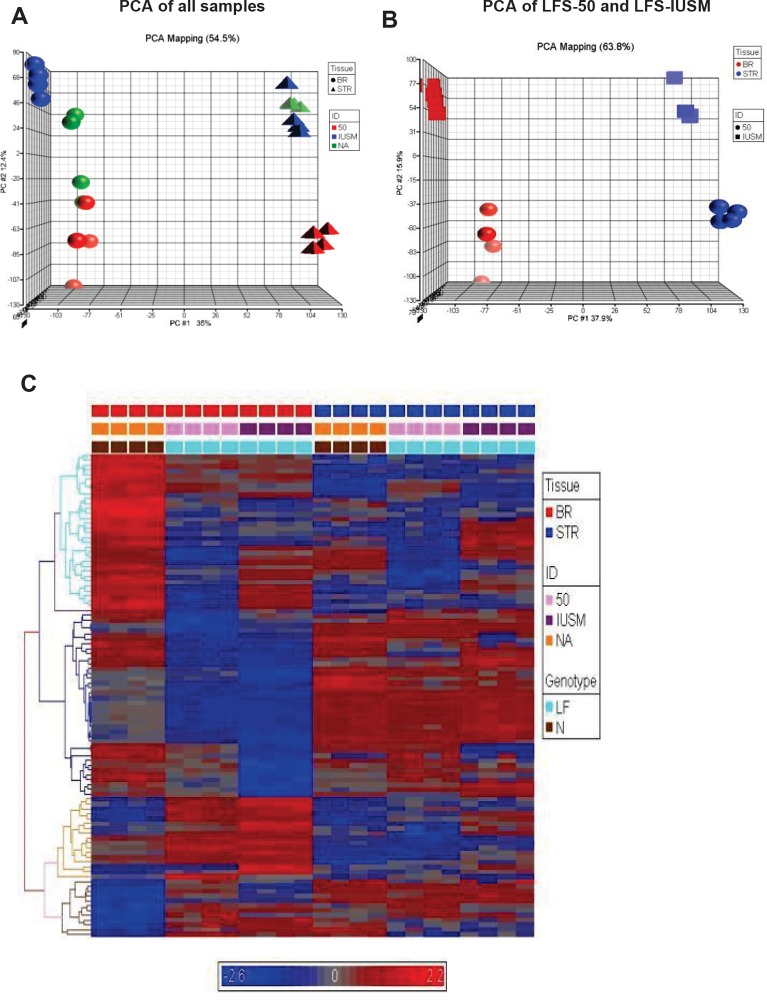
Gene expression patterns between TP53 heterozygous and WT breast epithelial and stromal cells. A) Principal component analyses (PCA) of samples. Spheres depict breast epithelial samples (BR), while triangles depict stromal samples. Red objects represent LFS patient 50 samples, blue objects represent samples derived from IUSM-LFS patient, and green objects represent normal/WT samples. B) PCA of LFS-50 compared to LFS-IUSM samples. C) Supervised heat-map with gene expression patterns of the top 100 genes noting clusters differentiating diseased vs. non-diseased samples. The bars above the panel depict sample clusters; top: tissue/sample type (left to right: BR, breast epithelial; STR, stromal); middle: ID/cell line (left to right: NA, normal/WT; 50, patient 50; IUSM, cells derived from IUSM patient); bottom: genotype/phenotype (left to right: N, normal/WT; LF, Li-Fraumeni syndrome). The different colored bars on the left of the panel represent different clusters of biological processes. Gene expression variation is depicted by color (red, up-regulated; blue, down-regulated; gray, no significant change). The genes and Gene Ontology of Biological Processes are listed in Supplemental Data.

Morphologically normal, breast-derived epithelial and stromal cells were established from TP53-haploinsufficient and mutation-negative (TP53 wild-type, WT) individuals. LFS is a rare disorder and the amount of breast tissue available from affected individuals in which to derive breast cell lines is therefore limited. One LFS sample (patient 50) was derived from the noninvolved tissue of a 31year-old female undergoing surgery for breast cancer. Patient 50 came from a family in which breast cancer and the *TP53* mutations were prevalent through at least three generations [14]. The other LFS (or LFS-like) sample (patient IUSM) was derived from the benign breast tissue of a 29-year old Caucasian female undergoing surgery for non-invasive ductal carcinoma and bilateral Paget's disease of the nipples. Patient IUSM also had a maternal aunt with bilateral breast cancer in her 30's and a male sibling with osteogenic sarcoma of a leg at age 13 who later died of a brain tumor at age 19. LFS patient 50 contained a heterozygous missense mutation in the DNA binding domain of *TP53* that affects the conformation of the p53, while the other sample (patient IUSM) had a heterozygous frameshift mutation in the proline-rich domain of *TP53*, resulting in a truncated protein. Four biologically independent replicates of these cells, and four biologically independent replicates from an age-matched female with no history of breast cancer, were used to analyze whole genome expression profiles of LFS heterozygous mutation-carrying and wild-type cells. Class comparison analyses (i.e., TP53 vs. WT) revealed notable changes in gene expression, suggesting that germline heterozygous TP53 mutations significantly alter the expression profiles of both primary epithelial cells and fibroblasts (Figure 1; Tables 1-3; Supplemental Figure 1; Supplemental Data File 1). The genes most differentially regulated in LFS vs. WT cells for both epithelial and fibroblast cell types are shown in Table 1.

**Table 1:** List of the top differentially regulated genes between both of the LFS and WT cells in epithelial and stromal tissue types.

Gene Symbol	Description	Fold Change	p-value
***Genes upregulated in LFS vs. WT epithelial cells***			
ZNF415	Zinc finger protein 415	10.411	5.96E-05
BIRC3	Baculoviral IAP repeat-containing 3	9.628	3.13E-03
NMES1	Normal mucosa of esophagus specific 1	9.509	1.43E-02
	Transcribed locus	9.431	1.35E-04
DNCI2	Dynein, cytoplasmic, intermediate polypeptide 2	7.322	5.94E-07
EGR3	Early growth response 3	7.059	5.23E-03
GPNMB	Glycoprotein (transmembrane) nmb	7.044	5.80E-04
ZNF506	zinc finger protein 506	7.005	8.66E-10
MICB	MHC class I polypeptide-related sequence B	6.998	1.23E-04
EMP2	epithelial membrane protein 2	6.809	6.28E-03
***Genes downregulated in LFS vs. WT epithelial cells***			
MYEF2	Myelin expression factor 2	−7.196	8.10E-06
DOC1	Downregulated in ovarian cancer 1	−8.229	1.25E-05
C7orf10	Chromosome 7 open reading frame 10	−10.124	1.86E-08
C13orf18	Chromosome 13 open reading frame 18	−10.519	6.49E-07
GHR	Growth hormone receptor	−11.471	7.45E-11
HOXB7	Homeo	−14.978	7.37E-08
ANGPTL4	Angiopoietin-like 4	−18.426	1.81E-09
SLC38A5	Solute carrier family 38, member 5	−18.469	2.92E-04
NEFL	Neurofilament, light polypeptide 68kDa	−19.512	8.72E-06
XG	Xg blood group (pseudoautosomal boundary-divided on the X chromosome)	−65.958	7.36E-05
***Genes upregulated in LFS vs. WT stromal cells***			
TM4SF1	Transmembrane 4 superfamily member 1	31.899	1.27E-05
TM4SF1	Transmembrane 4 superfamily member 1	29.644	5.12E-06
TM4SF1	Transmembrane 4 superfamily member 1	25.646	2.62E-05
TM4SF13	Transmembrane 4 superfamily member 13	12.675	2.54E-04
FABP5	Fatty acid binding protein 5 (psoriasis-associated)	10.582	3.50E-03
G0S2	Putative lymphocyte G0/G1 switch gene	10.572	3.60E-03
PTGS1	Prostaglandin-endoperoxide synthase 1 (prostaglandin G/H synthase and cyclooxygenase)	9.926	1.67E-05
RAMP	RA-regulated nuclear matrix-associated protein	9.155	1.13E-04
RAD51AP1	RAD51 associated protein 1	9.132	9.19E-03
FLJ31340	Hypothetical protein FLJ31340	9.020	2.03E-02
***Genes downregulated in LFS vs. WT stromal cells***			
ARHGAP26	Rho GTPase activating protein 26	−6.532	6.63E-08
CCND2	Cyclin D2	−6.615	1.17E-02
RGC32	Response gene to complement 32	−7.036	2.60E-16
PSG4	Pregnancy specific beta-1-glycoprotein 4	−7.149	1.09E-15
	Transcribed locus	−7.179	4.97E-10
RDH10	Retinol dehydrogenase 10 (all-trans)	−7.779	8.74E-12
STEAP2	Six transmembrane epithelial antigen of prostate 2	−8.158	1.67E-21
COPl	CARD only protein	−10.944	2.92E-08
GPM6B	Glycoprotein M6B	−11.299	8.01E-06
GPM6B	Glycoprotein M6B	−11.772	1.42E-06

**Table 2:** List of molecules within the top ten gene networks generated from IPA and significantly modulated between the LFS and WT breast epithelial or stromal cells.

Ingenuity Canonical Pathways	Molecules
***Epithelial Cells***	
Wnt/β-catenin Signaling	CSNK1E,CDKN2A,CSNK1G1,PPP2R2B,TGFB2,PPP2R2C,KREMEN1,DKK1,CTNNB1,EP300
Tight Junction Signaling	MYLK,MPDZ,CLDN12,PPP2R2B,PVRL3,TGFB2,PPP2R2C,ACTG2 (includes EG:72),CTNNB1
Cycle: G1/S CheckpointRegulation	CDKN2A,CCND2,NRG1,CDK6,TGFB2
Aryl Hydrocarbon ReceptorSignaling	CDKN2A,CCND2,ALDH1A3,NQO1,CDK6,TGFB2,IL1B,EP300
Coagulation System	PROS1,SERPINA1,PLAU,F3
NRF2-mediated OxidativeStress Response	FTL,NQO1,DNAJC1,AOX1,ACTG2 (includes EG:72),FKBP5,TXNRD1,EP300,EPHX1
p53 Signaling	CDKN2A,CCND2,THBS1,C12ORF5,CTNNB1,EP300
Acute Phase ResponseSignaling	FTL,FN1,IL1RN,IL1B,C5,SERPINA1,STAT3,IL1RAP
Selenoamino AcidMetabolism	SEPHS1,GGT1,AHCY
Eicosanoid Signaling	AKR1C3,PNPLA3,PTGS2,GGT1
***Stromal Cells***	
Aryl Hydrocarbon ReceptorSignaling	TP53,GSTM1,CCNE2,POLA1,GSTM3 (includes EG:2947),NQO1,BAX,CHEK1,CCNA2,GSTM2,CCND2,ALDH1A3,CDKN1A,GSTM4,IL1B,DHFR,CDK2,M CM7
Role of BRCA1 in DNA Damage Response	TP53,RAD51,RFC4,MSH2,CDKN1A,MSH6,RFC2,RBL1,RFC5,CHEK1
Pyrimidine Metabolism	TYMS,PRIM1,NME5,DCK,POLE2,POLA1,RRM2B,RRM2,REV3L,RFC5,CTPS,NME7,RRM1,POLD3,NP, POLA2,TK1
p53 Signaling	TP53,CCNG1,GADD45B,CCND2,RRM2B,CDKN1A,TNFRSF10B,BAX,CDK2,BIRC5,CHEK1,DRAM (includes EG:55332)
Cycle: G1/S CheckpointRegulation	TP53,CCNE2,CCND2,HDAC8,SUV39H1,CDKN1A,NRG1,RBL1,CDK2
Glutathione Metabolism	GPX3,GSTM1,TRHDE,GSTM2,GSTM3 (includes EG:2947),GPX1,GSTM4,G6PD,H6PD,GCLM
Purine Metabolism	PRIM1,ATP1B1,NME5,DCK,DDX39,POLE2,POLA1,RRM2B,RRM2,REV3L,RFC5,NME7,RRM1,RAD51, PRPS2,POLD3,PRPS1,NP,ADA,POLA2,PDE5A,ENPP2,AOX1,PPAT
Pentose PhosphatePathway	PRPS2,PRPS1,TKT,G6PD,H6PD,ALDOC
NRF2-mediated OxidativeStress Response	GSTM1,DNAJC9,GSTM3 (includesEG:2947),NQO1,GSTM2,RRAS2,SOD2,CAT,GSTM4,SQSTM1,AOX1,GCLM,ACTC1,PRKD1,FTH1
Histidine Metabolism	PRPS2,PRPS1,ALDH1A3,FTSJ1,MAOA

**Table 3:** List of Most Significantly, Differentially Regulated Genes Between LFS-50 and LFS-IUSM Epithelial Cells

GENE TITLE	GENE SYMBOL	P-VALUE	FDR	FOLDCHANGE (50/IUSM)
CYTOCHROME P450, FAMILY 1, SUBFAMILY B, POLYPEPTIDE 1	CYP1B1	5.15E-09	0.000112	−16.2203
PROTEIN-L-ISOASPARTATE (D-ASPARTATE) O- METHYLTRANSFERASE DOMAIN CONTAINING 1	PCMTD1	3.63E-08	0.000290	−7.33201
CHURCHILL DOMAIN CONTAINING 1	CHURC1	3.82E-07	0.001748	3.2
SMU-1 SUPPRESSOR OF MEC-8 AND UNC-52 HOMOLOG (C. ELEGANS)	SMU1	4.60E-07	0.001748	−1.81118
DISCOIDIN DOMAIN RECEPTOR TYROSINE KINASE 2	DDR2	7.62E-07	0.001869	−6.44987
LYR MOTIF CONTAINING 5	LYRM5	9.57E-07	0.001869	−2.96636
GLUCOCORTICOID INDUCED TRANSCRIPT 1	GLCCI1	9.88E-07	0.001869	−3.69182
KTEL (LYS-TYR-GLU-LEU) CONTAINING 1	KTELC1	1.14E-06	0.001869	−2.9365
PROTEIN KINASE, CAMP-DEPENDENT, CATALYTIC, BETA	PRKACB	1.19E-06	0.001869	−1.84956
ROD1 REGULATOR OF DIFFERENTIATION 1 (S. POMBE)	ROD1	1.24E-06	0.001869	1.94034
TRANSMEMBRANE PROTEIN 157	TMEM157	1.53E-06	0.001869	−2.16878
ZINC FINGER PROTEIN 655	ZNF655	1.64E-06	0.001869	−3.33563
INOSITOL HEXAPHOSPHATE KINASE 2	IHPK2	1.67E-06	0.001869	−2.662
REPLICATION INITIATOR 1	REPIN1	1.73E-06	0.001869	−14.9282
ZINC FINGER PROTEIN 430	ZNF430	1.80E-06	0.001869	−2.65347
GLUTATHIONE PEROXIDASE 7	GPX7	1.91E-06	0.001869	−5.19146
TRANSCRIPTION ELONGATION FACTOR A (SII)-LIKE 1	TCEAL1	1.92E-06	0.001869	−3.0577
CYTOCHROME P450, FAMILY 1, SUBFAMILY B, POLYPEPTIDE 1	CYP1B1	2.05E-06	0.001869	−13.6735
CARBOXYLESTERASE 2 (INTESTINE, LIVER)	CES2	2.18E-06	0.001869	−5.24574
HYPOTHETICAL PROTEIN LOC339400	LOC339400	2.25E-06	0.001869	−18.8473
CCR4-NOT TRANSCRIPTION COMPLEX, SUBUNIT 6-LIKE	CNOT6L	2.27E-06	0.001869	−2.04665
ZINC FINGER PROTEIN 605	ZNF605	2.31E-06	0.001869	−2.70276
CHROMOSOME 9 OPEN READING FRAME 61	C9ORF61	2.41E-06	0.001869	−8.82731
TP53 REGULATED INHIBITOR OF APOPTOSIS 1	TRIAP1	2.43E-06	0.001869	−2.65011
ZINC FINGER HOMEOBOX 4	ZFHX4	2.56E-06	0.001869	−4.08673

Principal component analyses (PCA) of the global expression profiles revealed that each sample set clustered together (Figure 1A). The stromal and epithelial samples were clearly positioned in two different coordinates from each other (Figure 1A). Interestingly, the LFS patient 50 epithelial samples were positioned separately from the other LFS and WT samples (Figure 1B). The large difference in the nature of the mutation and position of the LFS-50 samples from the other (LFS-IUSM) samples could affect the severity of haploinsufficiency of the *TP53* mutation. Hierarchical clustering of the top 100 genes from the arrays revealed distinct clusters differentiating disease genotype, the stromal and epithelial samples, as well as LFS and WT samples (Figure 1C; Supplemental Figure 1; Supplemental Data File 1). The epithelial samples revealed more distinct clustering of the top 100 genes between the LFS and WT individuals than the stromal samples.

To confirm the results of the gene expression microarray, qRT-PCR validation was performed on the RNA samples used for the initial array. A full list of the validated primers can be found in Supplemental Table 1. The genes examined represent a number of different functions in p53 signaling, cell proliferation (cell cycle regulation), and cell survival (apoptosis) as detected from the gene expression data and Ingenuity Pathway Analysis described below. The expressions of genes in LFS samples by qRT-PCR were observed to have consistent dysregulation between normal and LFS cells, and were similar to those changes by gene microarray (Supplemental Table 2).

### Gene expression profiles of single-hit LFS epithelial cells compared to WT epithelial cell cultures.

Further data mining revealed highly significant differences for epithelial cell comparisons. One of the most dramatic differences in gene expression between both of the LFS epithelial samples and WT epithelial cells was in the Xg blood group protein (−66 fold; p<0.0001; Table 1), a cell surface antigen [15]. In addition, there was a significant upregulation of BIRC3 (9.6 fold; p<0.003) in the LFS cells (Table 1). Furthermore, a significant upregulation of transcription factor EP300 (p300; 3.1 fold; P=7.5 × 10^−7^; FDR 9.29 × 10^−5^; see Supplemental Data Files 2 and 3) was observed for the LFS epithelial cells. Notably, Table 1 shows that two of the most highly up-regulated genes with extreme statistical significance (p<0.00001) were the zinc finger-containing transcription factors ZN415 (10.4 fold change; P=5.96 × 10^−5^; FDR 2.16 × 10^−3^) and ZN506 (7.0 fold change; P=8.66 × 10^−10^; FDR 7.22 × 10^−7^). These are zinc finger-containing transcription factors and, similar to p300, their upregulation is likely to reflect a compensatory effect of TP53 haploinsufficiency to regulate critical TP53 targets. Similarly notable was the downregulation of multiple members of the HOXB7 signal transduction pathway (Table 1), which is important for maintenance of cell differentiation [16]. There is evidence that HOXB7 is regulated by the extracellular matrix in mammary epithelial cell cultures [17]. The downregulation of HOXB7 in both of the LFS epithelial cells, compared to WT, suggests an important role for the surrounding tissue and stroma for epithelial cell growth regulation in LFS patients.

**Figure 2:**
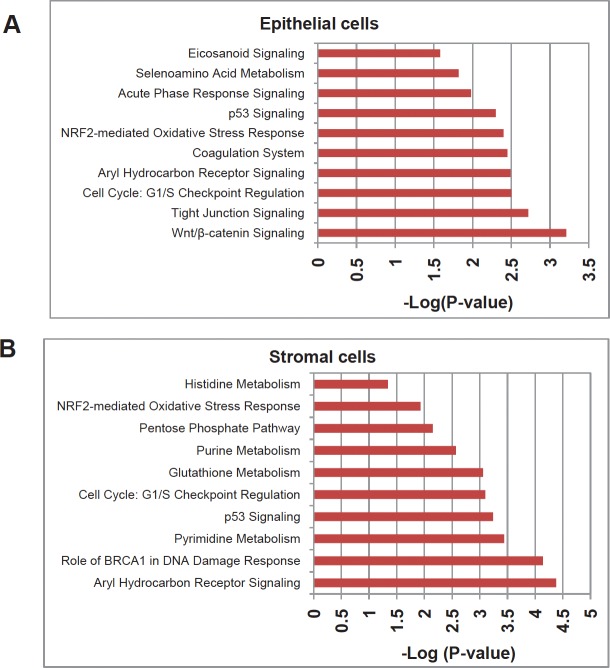
Top gene networks generated from IPA and significantly modulated (log p-value) between both of the LFS cells and WT cells. A) Top ten networks significantly modulated between LFS and WT breast epithelial cells. B) Top ten networks significantly modulated between LFS and WT breast stromal cells. Molecules associated within these pathways are listed in Table 4.

**Figure 3:**
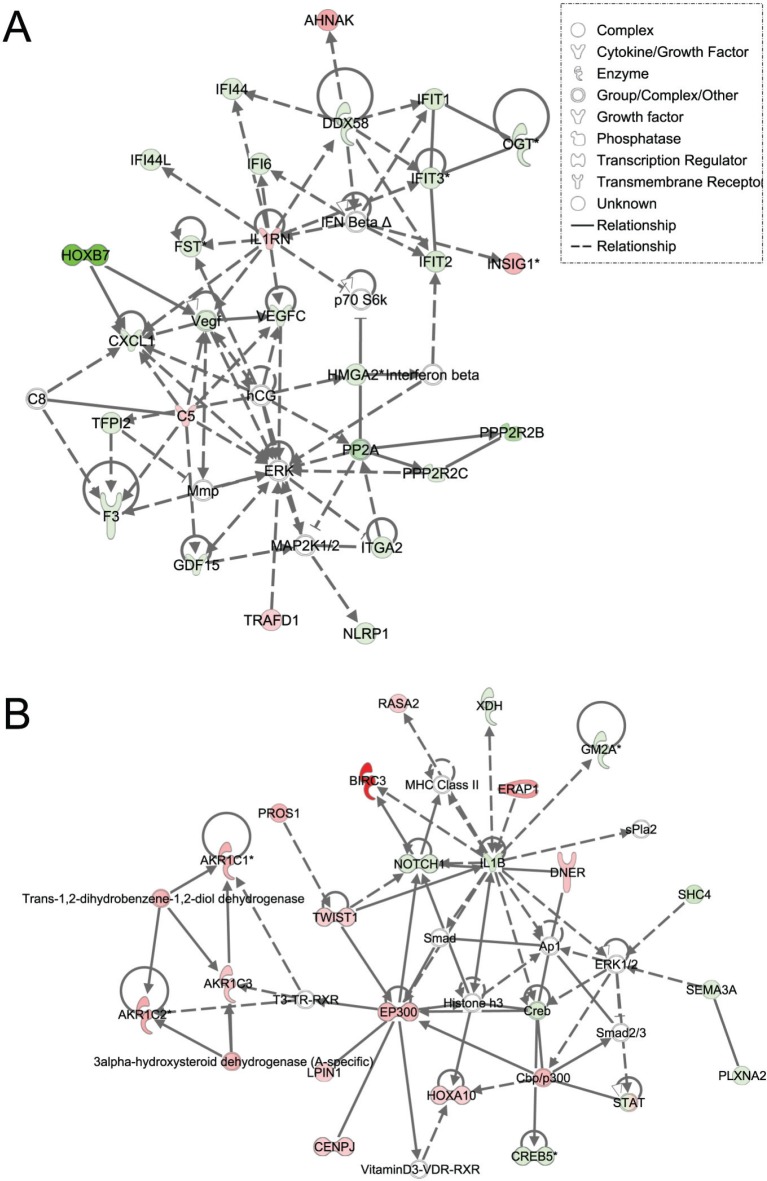
Ingenuity pathway analysis of genes differentially regulated in LFS vs. WT epithelial cells. Functional pathway analysis by IPA of ERK (A) and IL1B/p300/BIRC3 (B) genes and their interaction nodes in LFS breast epithelial cells relative to WT breast epithelial cells. Gene expression variation by at least 2-fold is depicted by color (red, up-regulated; green, down-regulated; gray, no significant change).

Using Ingenuity Pathway Analysis (IPA) with FDR of 10% and fold change cut-off of +/− 2, we evaluated the interaction and functional importance of the signaling pathways involving genes significantly dysregulated in both of the LFS epithelial cells compared to WT epithelial cells. The top ten canonical pathways that were significantly modulated between LFS and WT breast cells are depicted in Figure 2A. Molecules associated within these pathways are listed in Table 2. Significant pathways in the epithelial LFS vs. WT sample set included Wnt/β-catenin, tight junction, cell cycle, and oxidative stress signaling pathways. Noteworthy in these pathway analyses was a highly significant representation of the IPA-defined TP53 signaling pathway in the epithelial (-LogP value of 2.30) samples (Figure 2A; Supplemental Data File 4). Specific perturbations included the TP53 transcriptional network targets *CDKN2A, CCND2, THBS1, C12ORF5, CTNNB1*, and *EP300* (Table 2). Gene interaction networks analysis of the 472 genes differentially expressed in the TP53 haploinsufficient yet morphologically normal breast epithelial cell cultures revealed two significant networks relative to WT breast epithelial cells (Figure 3). Several genes were down-regulated in the *ERK* network (Figure 3A) and upregulated in the *IL1B/p300/BIRC3* (Figure 3B) in LFS breast epithelial cells relative to WT breast epithelial cells.

**Table 4:** List of Most Significantly, Differentially Regulated Genes Between LFS-50 and LFS-IUSM Stromal Cells

Ingenuity Canonical Pathways	Molecules
***Epithelial Cells***	
Wnt/β-catenin Signaling	CSNK1E,CDKN2A,CSNK1G1,PPP2R2B,TGFB2,PPP2R2C,KREMEN1,DKK1,CTNNB1,EP300
Tight Junction Signaling	MYLK,MPDZ,CLDN12,PPP2R2B,PVRL3,TGFB2,PPP2R2C,ACTG2 (includes EG:72),CTNNB1
Cycle: G1/S Checkpoint Regulation	CDKN2A,CCND2,NRG1,CDK6,TGFB2
Aryl Hydrocarbon Receptor Signaling	CDKN2A,CCND2,ALDH1A3,NQO1,CDK6,TGFB2,IL1B,EP300
Coagulation System	PROS1,SERPINA1,PLAU,F3
NRF2-mediated Oxidative Stress Response	FTL,NQO1,DNAJC1,AOX1,ACTG2 (includes EG:72),FKBP5,TXNRD1,EP300,EPHX1
p53 Signaling	CDKN2A,CCND2,THBS1,C12ORF5,CTNNB1,EP300
Acute Phase Response Signaling	FTL,FN1,IL1RN,IL1B,C5,SERPINA1,STAT3,IL1RAP
Selenoamino Acid Metabolism	SEPHS1,GGT1,AHCY
Eicosanoid Signaling	AKR1C3,PNPLA3,PTGS2,GGT1
***Stromal Cells***	
Aryl Hydrocarbon Receptor Signaling	TP53,GSTM1,CCNE2,POLA1,GSTM3 (includes EG:2947),NQO1,BAX,CHEK1,CCNA2,GSTM2,CCND2,ALDH1A3,CDKN1A,GSTM4,IL1B,DHFR,CDK2,MCM7
Role of BRCA1 in DNA Damage Response	TP53,RAD51,RFC4,MSH2,CDKN1A,MSH6,RFC2,RBL1,RFC5,CHEK1
Pyrimidine Metabolism	TYMS,PRIM1,NME5,DCK,POLE2,POLA1,RRM2B,RRM2,REV3L,RFC5,CTPS,NME7,RRM1,POLD3,NP,POLA2,TK1
p53 Signaling	TP53,CCNG1,GADD45B,CCND2,RRM2B,CDKN1A,TNFRSF10B,BAX,CDK2,BIRC5,CHEK1,DRAM (includes EG:55332)
Cycle: G1/S Checkpoint Regulation	TP53,CCNE2,CCND2,HDAC8,SUV39H1,CDKN1A,NRG1,RBL1,CDK2
Glutathione Metabolism	GPX3,GSTM1,TRHDE,GSTM2,GSTM3 (includes EG:2947),GPX1,GSTM4,G6PD,H6PD,GCLM
Purine Metabolism	PRIM1,ATP1B1,NME5,DCK,DDX39,POLE2,POLA1,RRM2B,RRM2,REV3L,RFC5,NME7,RRM1,RAD51,PRPS2,POLD3,PRPS1,NP,ADA,POLA2,PDE5A,ENPP2,AOX1,PPAT
Pentose Phosphate Pathway	PRPS2,PRPS1,TKT,G6PD,H6PD,ALDOC
NRF2-mediated Oxidative Stress Response	GSTM1,DNAJC9,GSTM3 (includes EG:2947),NQO1,GSTM2,RRAS2,SOD2,CAT,GSTM4,SQSTM1,AOX1,GCLM,ACTC1,PRKD1,FTH1
Histidine Metabolism	PRPS2,PRPS1,ALDH1A3,FTSJ1,MAOA

### Gene expression profiles of single-hit LFS stromal cells compared to WT stromal cell cultures.

An important strength of this study was the inclusion of a stromal cell array set to analyze gene expression profile changes between initiated LFS and WT cells. Thus, in addition to examining the breast epithelial cells of LFS vs. WT individuals, we were also able to identify gene expression alterations for the stromal fibroblast cells that may indicate changes in the breast microenvironment. These changes could predispose the p53-haploinsufficient epithelial cells to transformation. In examining the stromal cells from LFS vs. WT individuals (Table 1), there were striking differences in gene expression. The top four genes upregulated in LFS stromal cells were *TM4SF1* and *TM4SF13*. The gene products are members of the tetraspanin family of cell surface proteins. These proteins have been associated with cancer and are also known as tumor associated antigens [18]. *Cyclin D2* (*CCND2*), which is often lost in breast cancer due to promoter hypermethylation [19-20], was significantly downregulated in LFS stromal cells, compared to WT (6.6 fold; p<0.012), suggesting a marked dysregulation of the cell cycle in these stromal cells. Though cyclin D2 is involved in promoting the G1-S transition of the cell cycle, Meyyappan et al. [21] showed that this protein can also be growth-arresting, which may suggest why it was downregulated in breast stromal cells (Table 1). A similar yet less dramatic downregulation (1.9 fold; p<0.001) of cyclin D2 was observed in the LFS epithelial cells compared to WT epithelial cells (see Supplemental Data File 2). These findings indicate that loss of cyclin D2 is a very early event in cancer progression in the TP53 heterozygous breast epithelial and stromal cells. Another gene that was dysregulated in the LFS fibroblasts was the *G0S2* gene (10.5 fold upregulated; p<0.004; Table 1), which is involved in the G0 to G1 transition, leading to cell cycle activation [22]. The changes in expression of both *Cyclin D2* and *G0S2* indicate that the p53-haploinsufficient stromal cells have a substantial disruption of normal cell cycle progression, which suggests a role for these cells in breast tissue growth and therefore on cancer predisposition of LFS patients.

**Figure 4:**
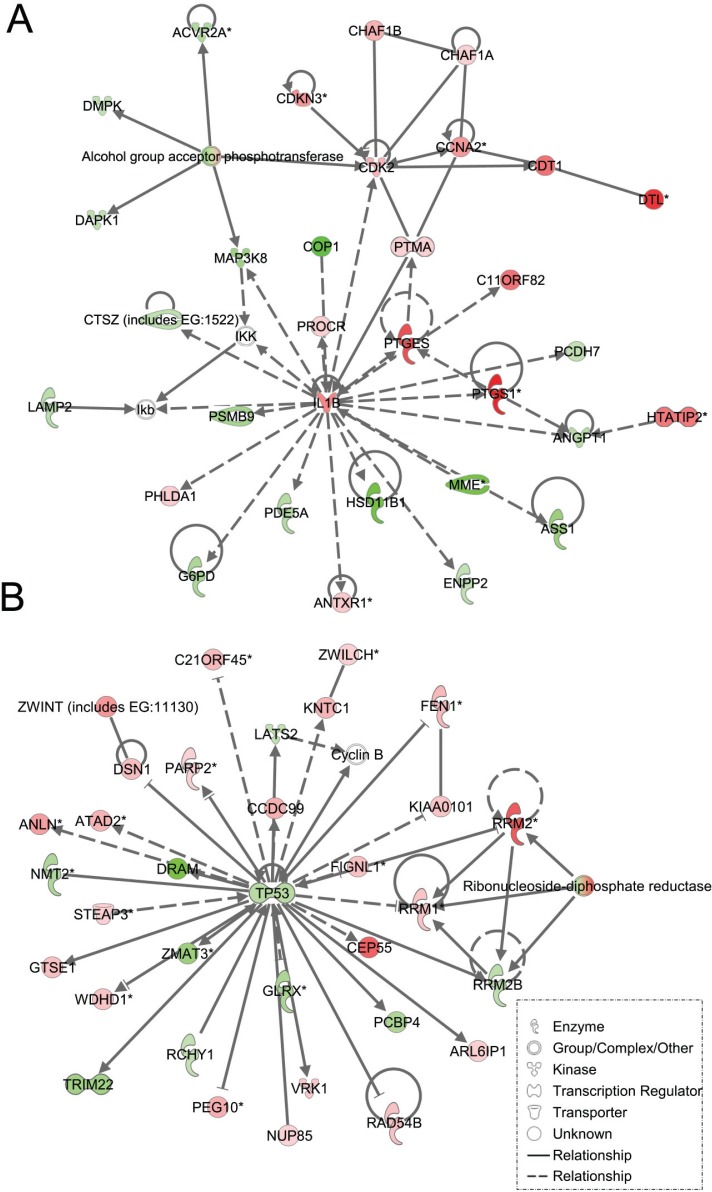
Ingenuity pathway analysis of genes differentially regulated in LFS vs. WT stromal cells. Functional pathway analysis by IPA of IL1B/CDK2 (A) and TP53 (B) gene pathways and their interaction nodes in LFS breast stromal cells relative to WT breast stromal cells.

As expected, the stromal fibroblast gene expression signature of TP53 haploinsufficiency was comprised of not only similar, but additional genes than epithelial cells in the IPA-defined TP53 signaling pathway (Figure 2B, Table 2). In addition, significant pathways in the stromal LFS vs. WT sample set included DNA damage response and amino acid metabolism. These data demonstrate and confirm in two different tissues that the genes involved in the TP53 pathway (-Log P value of 3.24) are especially susceptible to reductions in TP53 transcriptional activity. To corroborate this interesting result of significant network pathways, we performed IPA of stromal cells from LFS patients and matched normal subjects. Gene interaction networks analysis of the 1093 genes differentially expressed in the TP53 haploinsufficient, morphologically normal breast stromal cell cultures revealed two significant networks relative to WT breast stromal cells. These networks included the *IL1B/CDK2* (Figure 4A) and *TP53* (Figure 4B) nodes and their gene interactions. Interestingly, while *IL1B* was downregulated in the epithelial comparison set (Figure 3B) *IL1B* was upregulated in the stromal comparison set (Figure 4A).

Comparing the gene interactions of the epithelial and stromal LFS cell cultures to the WT samples, a significant interaction network contained *ERK*. Epithelial to mesenchymal transition (EMT) is an important pathway through which epithelial cells can progress to malignancy. Increased ERK signaling is one important pathway that contributes to EMT [23]. The genes within the *ERK* nodal network were depicted as mainly down-regulated and included *SULF1, MFGE8, LOXL1, LTBP1*, and *COL4A* (Suppl Figure 2A; Suppl Table 3). Also notable was the presence of alpha integrins and extracellular matrix proteins (e.g., laminins) that interact with other genes within this network. Furthermore, a second significant gene interaction network was the *NF-κB* interaction node (Suppl Figure 2B) where the upregulation of *BIRC3* and a downregulation of *GOS2* were also present.

### Differences in gene expression profiles between two LFS cell lines with different TP53 mutations

**Figure 5:**
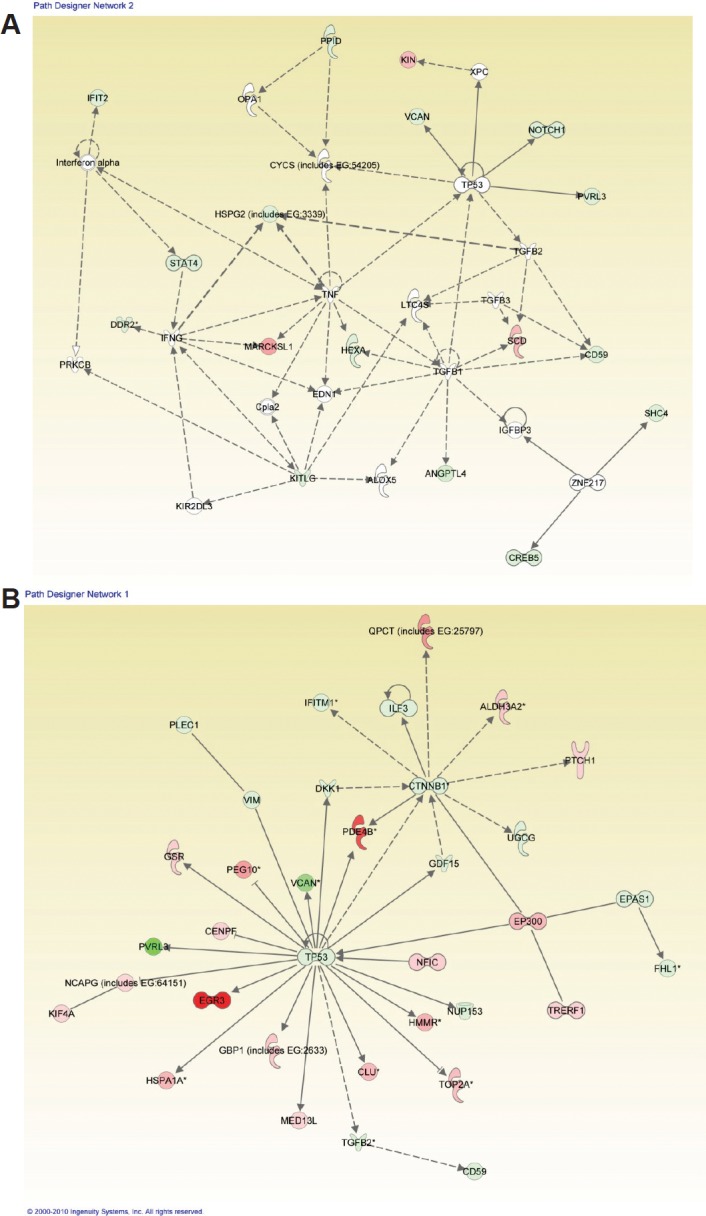
Ingenuity Pathway Analysis (IPA) for LFS-50 vs. WT Epithelial Cells and the Comparison of LFS-IUSM vs. WT Epithelial Cells. Functional pathway analysis by IPA of LFS-50 vs. WT Epithelial Cells (A) and LFS-IUSM vs. WT Epithelial Cells (B) gene comparisons and their interaction nodes.

When IPA was performed to distinguish comparisons of patient 50 vs. normal/WT epithelial samples as well as patient IUSM vs. WT epithelial samples, the top three canonical pathways were the same between the two sets of comparisons. Importantly, while the specific genes whose expression was altered were different between the two LFS patient samples (Figure 1 and Tables 3-4; Supplemental Figure 1C), the top networks and canonical pathways from IPA were similar. Thus, each LFS patient cells achieved essentially the same pathway alterations with slightly different granular details compared to WT samples (Figure 5). In summary, while the heterozygous p53 mutations in these two patients were different, the IPA results suggest that the phenotype of these cells derived from LFS or LFS-like patients are similar at the cellular level.

### Treatment of LFS breast epithelial cells with TP53 rescue drugs restores WT gene expression of altered genes.

**Figure 6:**
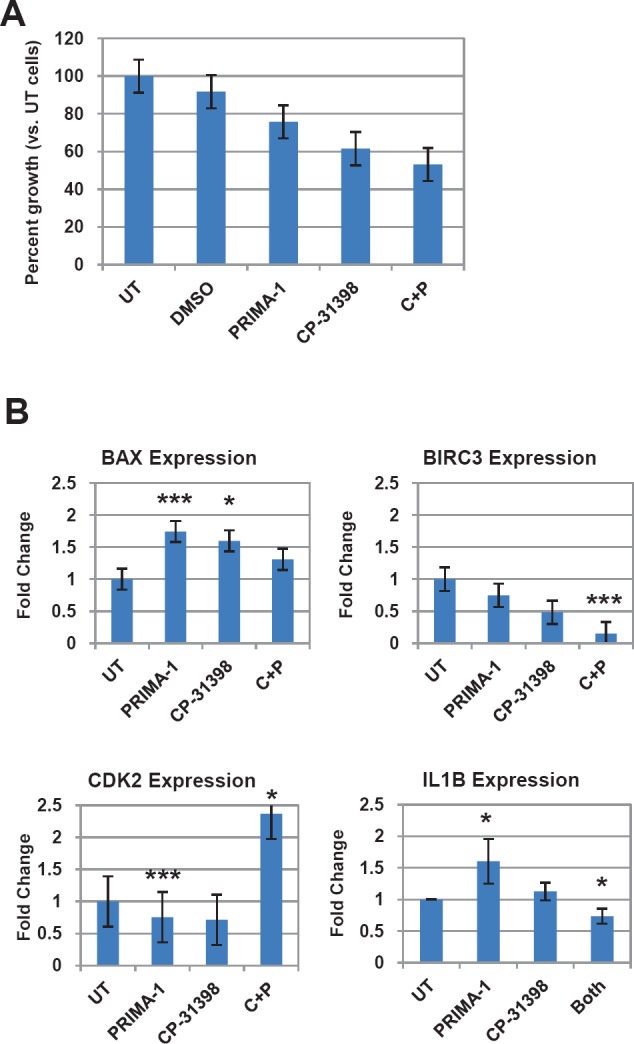
TP53 rescue agents restore expression of dysregulated genes in LFS breast epithelial cells. A) Effects on LFS-50 breast epithelial cell growth by the p53 rescue agents PRIMA-1 and CP-31398, compared to untreated control (normalized to 100%). B) Analysis of top genes (via qRT-PCR) from LFS microarray/p53 network of LFS epithelial cells (LFS-50 breast epithelial cells) treated with 10 μM PRIMA-1, CP-31398, or combination of both. Data is average of at least two independent experiments, with three replicates per treatment group, plus standard error. Statistical significance was determined by a two-tailed Students' t-test (MS Excel) where P<0.05 was considered significant (*, P<0.05; **, P<0.01; ***, P<0.001).

Since breast epithelial cells from LFS patient 50 contain a missense mutation in *TP53* that affects its protein conformation, we tested whether restoring p53 function by pharmacological agents will modulate expression of p53- and cell cycle-related genes (Supplemental Table 1). Cells were treated for 72 hours with 10 μM of PRIMA-1, CP-31398, or a combination of both, and compared to untreated cells. This dose was previously shown to induce senescence and reduce anchorage-independent growth on soft agar of tumorigenic LFS breast epithelial cells (Herbert et al., manuscript in preparation). Breast epithelial cells from LFS patient IUSM contain a frameshift mutation in the proline-rich domain of *TP53* and were not affected by treatment with the p53 rescue agents (data not shown). The combination of both p53 reactivating agents inhibited proliferation of LFS epithelial cells compared to untreated cells and to a greater extent than either drug alone (Figure 6A). Real-time RT-PCR was performed on the treated cells compared to untreated cells (Figure 6B). The genes investigated were the same p53/cell cycle gene sets as those investigated for the microarray validation of the LFS samples versus non-LFS samples (Suppl. Table 1). Of significant note, treatment of the LFS samples with the p53 rescue agents resulted in a reduction in the expression of the anti-apoptotic gene *BIRC3* in LFS samples compared to untreated samples. In this case, combination of PRIMA-1 and CP-31398 resulted in a significant reduction in *BIRC3* expression compared to either drug alone (P<0.001). As expected, treatment of LFS breast epithelial cells with PRIMA-1 or CP-31398 resulted in a significant increase in *BAX* gene expression (P<0.001), a pro-apoptotic gene, compared to untreated cells. Although combination of both agents did not result in an additive or synergistic fold increase in *BAX*, it was still greater than untreated samples, suggesting a possible saturation point of the pro-apoptotic gene. Treatment of LFS epithelial cells with PRIMA-1 restored the expression of *IL1B* (P=0.02); however, CP31398 nor the combination of both agents did not result in a statistically significant change compared to untreated samples. Furthermore, the combination of both agents actually was antagonistic to that of PRIMA-1 treatment alone (Figure 6B).

## DISCUSSION

In this study, we examined morphologically normal breast epithelial and stromal cells derived from patients (one classical LFS, the other with similar LFS clinical criteria as described in Mouchawar et al., ref. 24) with heterozygous mutations in *TP53* (“one-hit”) compared to cells derived from control (wild-type *TP53*) individuals. We observed significant differences in gene expression profiles between the wild-type cells and LFS cells for both cell types. Many of the differentially regulated genes were involved in signaling pathways known to be dysregulated in cancer, breast cancer in particular, including cell cycle regulation, apoptosis, and the WNT signaling pathway. Together, activation of these aberrant pathways is likely to contribute to cancer initiation in normal tissue of LFS patients.

The *TP53* tumor suppressor gene responds to a variety of cellular stressors, including DNA damage, hypoxia, metabolic stress, and oncogene activation. Under these conditions, the p53 protein is stabilized and initiates a transcriptional program resulting in DNA repair, cell-cycle arrest, senescence, or apoptosis. The specific program triggered is heterogeneous and depends on the type and strength of the incoming stress signals and the cellular context in which the response is executed. Although mutations affecting p53 are present in virtually all human cancers, “stress-induced” non-mutational activation of p53 occur very early in cancer progression and may precede and perhaps facilitate mutational activation associated with p53 [25-28]. In addition, recent evidence on early-onset breast cancers that did not meet the clinical criteria of LFS suggested that germline *TP53* mutations play a larger role in disease progression than previously considered [24].

Several additional players in stress response, apoptosis, and cell signaling shown here were noted for significant changes in gene expression and gene interaction networks. For example, we found changes in gene expression and networks for *BAX* and *IL1B* which are pro-apoptotic and pro-inflammatory response genes, respectively [29-30]. In addition, clustering analyses between LFS and WT samples highlighted *WISP3* which is an anti-inflammatory response gene which help prolong cell growth and survival [31]. Other significant genes in the analyses included *CDK2, CDKN1A* (p21), and *CHEK1*, all regulators of the cell cycle [32-34]. *BIRC3*, also known as cIAP2, plays an important role in promoting cell survival and inhibiting apoptosis [35]. The increased expression of *BIRC3* found in our analyses of LFS breast epithelial and stromal cells, and the normalization of expression with TP53 conformational rescue drugs, are consistent with an important role for BIRC3 anti-apoptotic signals in LFS initiated cells, and with recent findings that BIRC3 can drive tumorigenesis on a p53-deficient background in mouse osteosarcoma [36]. Importantly, the presence of the *BIRC3* signature suggests a potentially relevant early detection biomarker for TP53 haploinsufficiency that could facilitate cellular transformation in LFS pre-neoplastic cells, and be a potential chemopreventative drug target.

Also notable in our analyses was the upregulation of *EP300* (p300) in LFS samples compared to WT samples. p300 is a transcription factor in a number of pathways, including as a coactivator that competes with the coactivator CBP for TP53 binding and TP53 signaling [37]. At the same time, CBP mRNA levels were not significantly changed in these analyses (p>0.05). The upregulation of p300 transactivation likely reflects a compensatory effect of cells to stimulate TP53-p300 critical transcriptional genes in response to TP53 haploinsufficiency in breast epithelial and stromal cells. Furthermore, several zinc finger-containing transcription factors were also upregulated in our analyses and, similar to p300, their upregulation may suggest a compensatory effect of TP53 haploinsufficiency to regulate critical TP53 targets.

An important strength of our study was the inclusion of a stromal cell array set, in addition to breast epithelial cells, to analyze gene expression profile changes between LFS (heterozygous mutation in *TP53*) and WT stromal cells. Gene expression changes in the stromal compartment of one-hit LFS samples compared to wild-type samples may indicate changes in the breast microenvironment that play a role in cancer progression, including influences on epithelial to mesenchymal transition (EMT). Our IPA analyses shows that *IL1B* (*interleukin-1β* or *IL-1β*) represents a significant gene network and signaling pathway. We showed that *IL1B* was upregulated in the stromal LFS samples compared to WT samples. Because IL-1β is a key pro-inflammatory secreted cytokine that is cleaved by caspase-1 [38], it may be useful as a biomarker for morphologically normal, but molecularly abnormal, breast tissue. Additionally, these data form the basis for future studies that examine whether inhibition of IL-1β signaling plays a mechanistic role in preventing cancer progression and EMT in TP53-haploinsufficient cells. While most reports on *IL1B* are related to *H. pylori* infection and gastric cancer [39 for review] these studies have suggested that IL-1β is a critical link in inflammation as it leads to cancer [40]. Therefore, it will be important to examine the impact of *IL1B* expression as a biomarker of increased breast cancer risk in LFS patients.

Recently, p53 has been shown to function in aging and senescence through the regulation of mTOR, a key player in aging, metabolism and autophagy [41-44]. DNA repair deficiency diseases are classified as part of the group of metabolic syndromes. While there are no known relationships between LFS patients and metabolic syndrome symptoms (J. Fraumeni, personal communication), there is a current clinical trial on the “Role of p53 Gene in Metabolism Regulation in Patients with Li-Fraumeni Syndrome” (ClinicalTrials.gov Identifier: NCT00406445). Furthermore, in our study of one-hit *TP53* early passage, logarithmically growing cell lines, mTOR or associated genes were not significantly altered compared to the wild-type cells (stromal or epithelial). However, as the aging stroma has been shown to promote carcinogenesis [41, 45], the alterations in pathways involved with aging metabolism and inflammation observed in our study on one-hit *TP53* cells may support other studies demonstrating the role of p53 in these processes [41, 46-47]. In addition, these findings may offer an explanation, in part, for the early incidence of cancer in Li-Fraumeni syndrome families.

Genetically restoring p53 function alone, without additional treatments, has been shown to be sufficient to induce regression of advanced tumors [48-51]. Significantly, pharmacologic intervention by small molecules that rescue mutant p53 or activate wild-type protein can suppress or delay growth of established tumors in animals [52, 26]. Furthermore, we demonstrated that expression of a subset of these genes can be reverted to normal, wild-type individual levels by TP53 conformational modulators such as PRIMA-1 or CP-31398. In particular, the normalization of elevated *BIRC3* expression levels in a morphologically normal LFS cell line with these drugs suggests BIRC3 is a particularly attractive chemopreventative target. This defined gene expression panel can be used as potential biomarkers for TP53 conformation-modulating small molecules in the chemoprevention of TP53 mutation causing malignancies, both in LFS and in the general population, as TP53 mutations are common.

Families with Li-Fraumeni Syndrome are at high risk of dying from a variety of malignancies. The diversity of cancer sites makes intensive cancer surveillance particularly important for LFS mutation carriers. Individuals with BRCA1/2 or Lynch syndrome gene mutations have improved outcomes after diagnosis with intensive cancer surveillance. Individuals with LFS are even more likely to benefit from intensive cancer surveillance. Diagnosis of LFS is often difficult because many of the TP53 mutations are missense, which requires clinicians to have a very high level of evidence to make a positive diagnosis. It is therefore important to find distinctions between LFS and normal tissue to facilitate early diagnosis and targeted chemoprevention. Our data defined a potential molecular diagnostic tool that can be used to increase the depth of molecular testing used to confirm that an individual carries a LFS-causing pathological TP53 missense mutation. Our data also defined sets of genes in two different tissues that are especially susceptible to small decreases, or “one-hit” effects, in TP53 levels [9]. Significantly, our study suggests that the nature and site of mutations in the p53 underwrite the severity of abnormal molecular changes in the context of the LFS syndrome. These findings are supported by the recent studies which showed that subtle variations in *Pten* copy number determine cancer susceptibility in mouse models [11]. Further studies will be important to test whether these genes may be useful for distinguishing between LFS and sporadic TP53-deficient tissues from normal tissues in individuals at risk of cancer. In summary, because breast cancer incidence in LFS cohorts is very high [53], comparing abnormal pathways in LFS with those abnormal pathways in other inherited deficiencies, such as BRCA1/2 or Cowden syndrome which also predispose individuals to breast cancer, in addition to examining sporadic breast cancer, might further our understanding of treatment for breast cancer.

## MATERIALS AND METHODS

### Ethics Statement

Informed consent was obtained to collect patient tissue and this research was conducted according to the ethical standards and principles expressed in the Declaration of Helsinki. Ethical approval was obtained from the local research ethics committee in compliance with HIPAA privacy regulations as well as Institutional Review Board regulations governing patient-oriented research (IRB protocol #0403-87).

### Patient Details, Tissue Procurement, and Cell Culture

An LFS-like series was derived in 2006 from benign breast tissue of a 29-year old white female with non-invasive ductal carcinoma and bilateral Paget's disease of the nipples undergoing surgery at Indiana University School of Medicine (IUSM). Human breast tissue was minced or enzymatically digested with collagenase I, plated onto culture dishes and cultured in defined media to select for human mammary epithelial (HME) or stromal (HMS) cells as previously described [14]. A heterozygous *TP53* 12141delG germline frameshift mutation was identified in both the epithelial and stromal cells by conventional sequencing of exons 2-11 and intron-exon boundaries (Herbert, unpublished observations). The HME/HMS50 cell series (a generous gift by J.W. Shay) was derived from a 31-year-old Li-Fraumeni syndrome (LFS) patient's benign breast tissue (containing a heterozygous germline mutation at codon 133 in exon 5 in one of the two alleles of the *TP53* gene (Met to Thr [M133T]) that affects wild-type p53 protein conformation) as previously characterized [14]. Normal human mammary epithelial and stromal cells (a generous gift by J.W. Shay) derived from an age-matched female with no history of cancer were cultured as previously described [14]. The cell lines have been tested for *TP53* mutations by conventional sequencing, as well as characterization of cell surface markers and mycoplasma by immunocytology or thermocycler, within the last year and authenticated to have the same mutations, characteristics, and were mycoplasma-free, respectively.

HME cells were cultured in modified basal medium 171 (Cascade Biologics, Portland, OR) supplemented with 0.5% bovine pituitary extract (Hammond Cell Technologies), 100 μg/ml epidermal growth factor (Invitrogen), 10 μg/ml insulin, 1 μg/ml hydrocortisone, 10 μg/ml transferrin (Sigma-Aldrich, St. Louis, MO). Medium was changed every 2-3 days. HMS cells were cultured as described [14]. All cells were tested at the same log phase of cell growth and at similar passages.

### RNA Extraction and Preparation

Four biologically independent samples for each experimental group were collected for RNA, according to the Center for Medical Genomics guidelines [54]. Total RNA was prepared from cultured cells using the Qiagen RNeasy kit. All RNA samples were confirmed to have an A260/280 ratio of >1.8 by spectrophotometer and gel electrophoresis. Total RNA was diluted to a concentration of 1 μg/μl and 10 μg was given the Center for Medical Genomics for microarray processing. RNA integrity was further validated on an Agilent Bioanalyzer. All the samples showed distinct peaks corresponding to intact 28S and 18S ribosomal RNAs and therefore were included in the analysis.

### Microarray Processing and Analysis

Microarray processing was performed at the Center for Medical Genomics at the Indiana University School of Medicine. Preparation of cDNA and cRNA, as well as labeling was carried out according to the protocols recommended by Affymetrix in the GeneChip® Expression Analysis Technical Manual (Affymetrix, Santa Clara, CA). Arrays (HGU133 plus 2.0) were hybridized for 17h at 42^o^C. The arrays were washed and stained protocol by fluidics stations controlled by GCOS software using the standard Affymetrix protocol. The microarrays were scanned using a dedicated Model 3000 scanner controlled by GCOS software. The average intensity on each array was normalized by global scaling to a target intensity of 1000. Data were extracted using the Affymetrix Microarray Suite 5 (MAS5) algorithm and exported for analysis. Expression Data were deposited into the Gene Expression Omnibus (GSE accession #GSE23994).

The MAS5 data were filtered to eliminate any gene that was not called present in at least 50% of the samples in at least one group [55]. Data was log base 2 transformed and ANOVA (Analysis of Variance) was performed using the log transformed data. False discovery rate (FDR) was calculated using the Benjamini and Hochberg method [54]. Partek Genomics Suite software (Partek, Inc. St. Louis, MO) was used for hierarchical clustering. Log transformed data for the top 200 genes, as determined by p-value from the ANOVA, were clustered using Pearson's Dissimilarity as the distance measure and average linkage. The arrays were left unclustered.

### Ingenuity Pathway Analysis of Gene Expression Arrays

Ingenuity Pathway Analysis was executed on a subset of the original microarray data. Microarray output includes a value (‘P’, ‘M’, or ‘A’) for each transcript describing the confidence of detection. We filtered out transcripts that did not show sufficient read status. We required that a gene be present in at least half of the samples in at least one of four groups within the experiment: epithelial diseased, epithelial normal, stromal diseased or stromal normal [55]. This limited the original 54675 mRNAs to 21684.

The analysis for this study was generated using Partek^®^ software (Partek Inc., St. Louis, MO, USA) to calculate p-value and fold change for each logical comparison (epithelial diseased versus epithelial non-diseased, stromal diseased versus stromal non-diseased, and the differences between the epithelial comparison versus the stromal comparison). The p-value was calculated using a mixed-effect model with disease and cell type as two factors (or ‘fixed effects’) and cell line as a ‘random effect’. Next, we approximated a false discovery rate (FDR) score for each protein using the Benjamini-Hochberg method [56-57]. Finally, we limited the data set for IPA analysis to genes having a FDR score less than or equal to 0.1 (meaning an overall FDR for the entire data set of 10%) and an absolute fold change greater than or equal to 2 (meaning fold change must be greater than 2 or less than −2). At this point we separated the data set into logical analysis groups.

The molecular interactions among differentially-expressed genes (FDR≤0.1) were investigated using Ingenuity Pathway Analysis (IPA 6.1; Ingenuity systems, www.ingenuity.com; Mountain View, CA). Each gene identifier was mapped to its corresponding gene in the Ingenuity Pathway Knowledge Base (IPKB). These genes were overlaid onto a global network developed from the information contained in the IPKB. Networks of these genes, defined as the reflection of all interactions of a given gene defined in the literature, were then algorithmically generated based on their connectivity.

### Real-Time RT-PCR validation of microarray analysis

Validation of the microarray results were conducted by real-time RT-PCR using primers to a subset of genes from the microarray (Supplemental Table 1). Analysis was performed twice using triplicate repeats of RNA from each cell type and disease state. Analysis was performed using a 7500 PCR system and the corresponding 7500 SDS software (Applied Biosystems, Foster City, CA). For each gene, the threshold cycle number (Ct) was determined for all samples and individual sample Ct's were normalized to those of the housekeeping gene β-actin. Relative gene expression changes were quantified by exporting raw Ct values to MS Excel for ΔΔCt analysis and fold-change (2^(-ΔΔCt)) compared to WT or untreated control samples.

### Treatment with TP53 rescue agents

LFS breast epithelial cells were plated in 6-well dishes in the absence or presence of CP-31398 (N'-{2-[2-(4-Methoxy-phenyl)-vinyl]-quinazolin-4-yl}-N,N-dimethyl-propane-1,3-diamine hydrochloride) or PRIMA-1 (p53 reactivation and induction of massive apoptosis; 2,2-Bis(Hydroxymethyl)-3-Quinuclidinone) at different concentrations compared to untreated and solvent controls. CP-31398 and PRIMA-1 were supplied by DCP Repository/Fisher BioServices (Germantown, MD) and were dissolved in DMSO at 10 mM stock concentrations. A preliminary cytotoxicity test was performed to determine the highest nontoxic dose to be tested as well as EC50 (GraphPad Prism analysis). After 72 hr of treatment, cells were collected and used for gene expression analyses by real-time PCR described above. Statistical significance was determined by a two-tailed Students' t-test where P<0.05 was considered significant.

## 

Suppl. Figure 1 A-B) Supervised heat-map with gene expression patterns of the top 100 genes noting clusters differentiating diseased vs. non-diseased epithelial samples (A) and stromal samples (B). C) Unsupervised heat-map with gene expression patterns of the top 100 genes. The bars above the panel depict sample clusters; top: tissue/sample type (left to right: BR, breast epithelial; STR, stromal); middle: ID/cell line (left to right: NA, normal/WT; 50, patient 50; IUSM, cells derived from IUSM patient); bottom: genotype/phenotype (left to right: N, normal/WT; LF, Li-Fraumeni syndrome). The different colored bars on the left of the panel represent different clusters of biological processes. Gene expression variation is depicted by color (red, up-regulated; blue, down-regulated; gray, no significant change). The genes and Gene Ontology of Biological Processes is listed in Supplemental Data.

Suppl. Figure 2 **Ingenuity Pathway Analysis (IPA) Between the Interaction of LFS and WT Cells with the Interaction of Epithelial and Stromal Cells.** Functional pathway analysis by IPA of IL1B/CDKN2A (A) and ERK (B) genes and their interaction nodes in epithelial cells relative to stromal cells for genes that differed between the LFS vs. WT phenotypes.
