# Supplementary material for: A molecular signature of normal breast epithelial and stromal cells from Li-Fraumeni syndrome mutation carriers
Source: Oncotarget. 2010 Oct 6;1(6):405–22. doi: 10.18632/oncotarget.175 (PMC3039408; doi:10.18632/oncotarget.175)
Supplement: Supplementary file 3 [file oncotarget-01-405-s003.docx]

**Suppl. Table 1.**

| **gene** | **forward primer sequence (5'-3')** | **conc. (nM)** | **reverse primer sequence (5'-3')** | **conc. (nM)** | **TM** |
| --- | --- | --- | --- | --- | --- |
| BAX | CCGCCGTGGACACAGACT | 400 | TTTGACCACGAGTTCCGGG | 400 | 84.2 |
| BIRC3 | TGTGGGTAACAGTGATGATG | 200 | TGAACTTGACGGATGAACTC | 300 | 79.1 |
| CDK2 | CTCCTCCAGTGTGGGCTTGA | 300 | ACTTTCCCCCCTTGGTCAC | 100 | 81.9 |
| CDKN1A | GCGGCAGACCAGCATGAC | 200 | GTTTCCGGGCGAGATGTAGA | 300 | 84.7 |
| CHEK1 | TTGGGCTATCAATGGAAGAAA | 200 | TCATCCATTTCTAACAAATTCACTT | 300 | 73.3 |
| EP300 | GACCCTCAGCTTTTAGGAATCC | 200 | TGCCGTAGCAACACAGTGTCT | 200 | 80.2 |
| IL1B | CAGAGAGTCCTGTG CTGAAT | 100 | GTAGGAGAGGTC AGAGAGGC | 300 | 83.2 |
| P53 | GCC TGA GGT TGG CTC TGA CT | 200 | CCC ATG CAG GAA CTG TTA CAC A | 300 | 77.9 |
