# Supplementary material for: A molecular signature of normal breast epithelial and stromal cells from Li-Fraumeni syndrome mutation carriers
Source: Oncotarget. 2010 Oct 6;1(6):405–22. doi: 10.18632/oncotarget.175 (PMC3039408; doi:10.18632/oncotarget.175)
Supplement: Supplementary file 4 [file oncotarget-01-405-s004.doc]

**Supplemental Table 2.**

| ***LFS50 vs. WT comparison (epithelial cells)*** | |  |  |
| --- | --- | --- | --- |
| **gene symbol** | **description** | **Affymetrix** | **qRT-PCR** |
| BAX | BCL2-associated X protein | -1.89 | -2.01 |
| BIRC3 | Baculoviral IAP repeat-containing 3 | 17.96 | 37.03 |
| CDK2 | Cyclin-dependent kinase 2 | 2.25 | 1.29 |
| CDKN1A | Cyclin-dependent kinase inhibitor 1A (p21, Cip1) | -1.83 | -1.24 |
| CHEK1 | CHK1 checkpoint homolog (S. pombe) | 2.26 | 1.09 |
| EP300 | E1A binding protein p300 | 1.99 | 1.74 |
| IL1B | Interleukin 1, beta | -2.96 | -1.17 |
| ***LFS-IUSM vs. WT comparison (epithelial cells)*** | |  |  |
| **gene symbol** | **description** | **Affymetrix** | **qRT-PCR** |
| BAX | BCL2-associated X protein | -1.36 | -2.41 |
| BIRC3 | Baculoviral IAP repeat-containing 3 | 1.30 | 1.57 |
| CDK2 | Cyclin-dependent kinase 2 | -1.34 | -5.18 |
| CDKN1A | Cyclin-dependent kinase inhibitor 1A (p21, Cip1) | -1.22 | -1.20 |
| CHEK1 | CHK1 checkpoint homolog (S. pombe) | -1.13 | -7.47 |
| EP300 | E1A binding protein p300 | 2.64 | 3.30 |
| IL1B | Interleukin 1, beta | -6.61 | -9.28 |
| ***LFS50 vs. WT comparison (stromal cells)*** | |  |  |
| **gene symbol** | **description** | **Affymetrix** | **qRT-PCR** |
| BAX | BCL2-associated X protein | -2.38 | -1.80 |
| BIRC3 | Baculoviral IAP repeat-containing 3 | 3.01 | 5.91 |
| CDK2 | Cyclin-dependent kinase 2 | 2.69 | 14.77 |
| CDKN1A | Cyclin-dependent kinase inhibitor 1A (p21, Cip1) | -10.85 | -2.41 |
| CHEK1 | CHK1 checkpoint homolog (S. pombe) | 2.31 | 1.29 |
| EP300 | E1A binding protein p300 | -1.12 | -2.70 |
| IL1B | Interleukin 1, beta | 9.40 | 180.68 |
| ***LFS-IUSM vs. WT comparison (stromal cells)*** | |  |  |
| gene symbol | description | Affymetrix | qRT-PCR |
| BAX | BCL2-associated X protein | -3.61 | -1.97 |
| BIRC3 | Baculoviral IAP repeat-containing 3 | -1.59 | -2.78 |
| CDK2 | Cyclin-dependent kinase 2 | 2.54 | 11.50 |
| CDKN1A | Cyclin-dependent kinase inhibitor 1A (p21, Cip1) | -2.81 | -1.24 |
| CHEK1 | CHK1 checkpoint homolog (S. pombe) | 1.53 | 1.60 |
| EP300 | E1A binding protein p300 | -1.09 | -1.50 |
| IL1B | Interleukin 1, beta | 1.75 | 1.36 |
