# Supplementary material for: A molecular signature of normal breast epithelial and stromal cells from Li-Fraumeni syndrome mutation carriers
Source: Oncotarget. 2010 Oct 6;1(6):405–22. doi: 10.18632/oncotarget.175 (PMC3039408; doi:10.18632/oncotarget.175)
Supplement: Supplementary file 5 [file oncotarget-01-405-s005.doc]

**Suppl Table 3**.

| **gene symbol** | **description** | **fold change** | **p-value** |
| --- | --- | --- | --- |
| ***Genes differentially upregulated between LFS vs. WT epithelial cells vs. fibroblast cells*** | | | |
| STEAP2 | Six transmembrane epithelial antigen of prostate 2 | 5.742 | 7.36E-19 |
| RDH10 | Retinol dehydrogenase 10 (all-trans) | 5.530 | 2.00E-09 |
| GALNTL2 | UDP-N-acetyl-alpha-D-galactosamine:polypeptide N-acetylgalactosaminyltransferase-like 2 | 5.422 | 2.77E-12 |
| HSD11B1 | Hydroxysteroid (11-beta) dehydrogenase 1 | 5.412 | 8.74E-13 |
| BIRC3 | Baculoviral IAP repeat-containing 3 | 5.230 | 3.47E-02 |
| EGR3 | Early growth response 3 | 5.208 | 3.15E-02 |
| GPM6B | Glycoprotein M6B | 5.032 | 8.01E-06 |
| MME | Membrane metallo-endopeptidase | 4.811 | 4.22E-12 |
| GPM6B | Glycoprotein M6B | 4.760 | 3.47E-06 |
| PSG4 | Pregnancy specific beta-1-glycoprotein 4 | 4.556 | 1.99E-12 |
| ***Genes differentially downregulated between LFS vs. WT epithelial cells vs. fibroblast cells*** | | | |
| COL4A1 | Collagen, type IV, alpha 1 | -4.983 | 4.14E-02 |
| C13orf18 | Chromosome 13 open reading frame 18 | -5.198 | 7.17E-05 |
| TFPI2 | Tissue factor pathway inhibitor 2 | -5.230 | 1.68E-06 |
| TCEAL7 | Transcription elongation factor A (SII)-like 7 | -5.365 | 4.29E-05 |
| PPP2R2B | Protein phosphatase 2 (formerly 2A), regulatory subunit B (PR 52), beta isoform | -6.000 | 3.95E-07 |
| PTGS1 | Prostaglandin-endoperoxide synthase 1 | -7.177 | 1.32E-03 |
| PTGS1 | Prostaglandin-endoperoxide synthase 1 | -7.538 | 7.45E-04 |
| GHR | Growth hormone receptor | -7.841 | 4.33E-10 |
| G0S2 | Putative lymphocyte G0/G1 switch gene | -9.724 | 1.41E-02 |
| NEFL | Neurofilament, light polypeptide 68kDa | -16.395 | 3.77E-04 |
